# PirB regulates asymmetries in hippocampal circuitry

**DOI:** 10.1371/journal.pone.0179377

**Published:** 2017-06-08

**Authors:** Hikari Ukai, Aiko Kawahara, Keiko Hirayama, Matthew Julian Case, Shotaro Aino, Masahiro Miyabe, Ken Wakita, Ryohei Oogi, Michiyo Kasayuki, Shihomi Kawashima, Shunichi Sugimoto, Kanako Chikamatsu, Noritaka Nitta, Tsuneyuki Koga, Ryuichi Shigemoto, Toshiyuki Takai, Isao Ito

**Affiliations:** 1 Department of Biology, Faculty of Science, Kyushu University, Fukuoka, Japan; 2 Institute of Science and Technology Austria (IST Austria), Klosterneuburg, Austria; 3 Department of Experimental Immunology, Institute of Development, Aging and Cancer, Tohoku University, Sendai, Japan; Nathan S Kline Institute, UNITED STATES

## Abstract

Left–right asymmetry is a fundamental feature of higher-order brain structure; however, the molecular basis of brain asymmetry remains unclear. We recently identified structural and functional asymmetries in mouse hippocampal circuitry that result from the asymmetrical distribution of two distinct populations of pyramidal cell synapses that differ in the density of the NMDA receptor subunit GluRε2 (also known as NR2B, GRIN2B or GluN2B). By examining the synaptic distribution of ε2 subunits, we previously found that β2-microglobulin-deficient mice, which lack cell surface expression of the vast majority of major histocompatibility complex class I (MHCI) proteins, do not exhibit circuit asymmetry. In the present study, we conducted electrophysiological and anatomical analyses on the hippocampal circuitry of mice with a knockout of the paired immunoglobulin-like receptor B (PirB), an MHCI receptor. As in β2-microglobulin-deficient mice, the PirB-deficient hippocampus lacked circuit asymmetries. This finding that MHCI loss-of-function mice and PirB knockout mice have identical phenotypes suggests that MHCI signals that produce hippocampal asymmetries are transduced through PirB. Our results provide evidence for a critical role of the MHCI/PirB signaling system in the generation of asymmetries in hippocampal circuitry.

## Introduction

**Fig 1:**
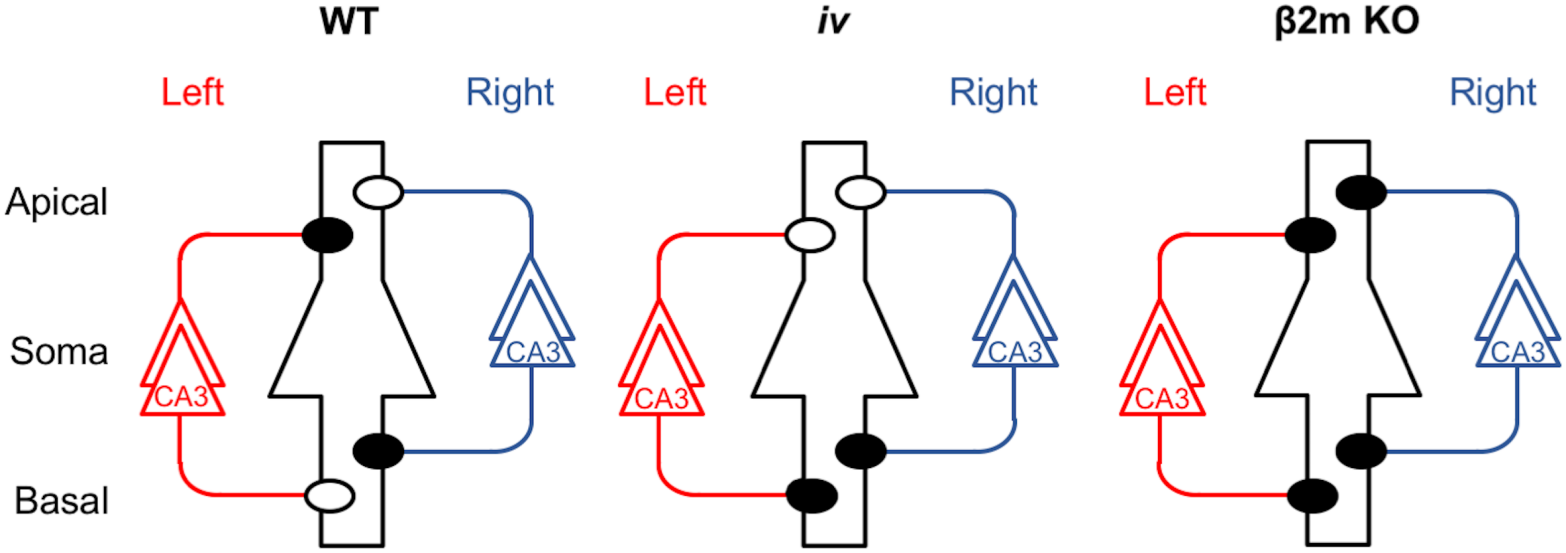
Hippocampal asymmetry and abnormalities in the *iv* and β2m KO mice. Left and right CA3 pyramidal neurons and their axons are colored red and blue, respectively. A postsynaptic CA1 pyramidal neuron is in the center, outlined in black, and it represents postsynaptic neurons in both left and right hemispheres. Closed and open circles represent ε2-dominant and ε2-non-dominant synapses, respectively. Apical, apical dendrites; Basal, basal dendrites; WT, wild-type; KO, knockout.

Left–right (L–R) asymmetry in brain structure and function is a central topic in neuroscience, and recent studies have identified possible molecular correlates of such asymmetry in the mouse hippocampus [1–3]. In the wild type (WT) mouse hippocampus, glutamatergic excitatory synapses formed between pyramidal neurons are classified into two distinct populations, ε2-dominant and ε2-non-dominant synapses, which differ in the density of synaptic NMDA receptor (NMDAR) ε2 (also known as NR2B, GRIN2B or GluN2B) subunits [1,2]. The ε2-dominant synapses are smaller and have a higher density of ε2 subunits than ε2-non-dominant synapses [4]. Therefore, NMDAR-mediated excitatory postsynaptic currents (NMDA EPSCs) in ε2-dominant synapses show higher sensitivity to Ro 25–6981, an ε2 subunit-selective antagonist [5–7], than those in ε2-non-dominant synapses [1,2]. In addition, the postnatal developmental establishment of long-term potentiation (LTP) in ε2-dominant synapses occurs earlier than in ε2-non-dominant synapses [1]. Furthermore, while ε2-non-dominant synapses show significant long-term depression (LTD) in response to low-frequency stimulation (1 Hz), ε2-dominant synapses do not [8]. These two populations of synapses are located asymmetrically in hippocampal circuitry, depending on the hemispheric origin of presynaptic inputs (referred to as L–R asymmetry) and on the cell polarity of the postsynaptic neuron (referred to as apical–basal, or A–B, asymmetry; Fig 1, WT).

We found two distinct mouse strains with defects in hippocampal asymmetry. The first is the *inversus viscerum* (*iv*) mouse [9,10], which is a spontaneous mutant with randomized laterality of the visceral organs [11–13]. Fifty percent of *iv* homozygous (*iv/iv*) mice exhibit reversed asymmetry (*situs inversus*), whereas the remaining are normal (*situs solitus*). However, the *iv* mouse hippocampus lacks L−R asymmetry, although it exhibits right isomerism (bilateral right-sidedness) in the synaptic distribution of the ε2 subunit, irrespective of the laterality of the visceral organs (Fig 1, *iv*) [14]. The second strain with defects in hippocampal asymmetry is the β2-microglobulin (β2m)-deficient mouse. β2m protein is a small subunit of major histocompatibility complex class I (MHCI) [15], and β2m-deficient mice are defective in stable cell surface expression of MHCI [16–20]. The β2m-deficient hippocampus lacks ε2-non-dominant synapses and therefore possesses ε2-dominant synapses only, resulting in a total loss of circuit asymmetry (Fig 1, β2m KO) [8]. Both *iv* and β2m-deficient mice exhibit deficits in retention of working memory [21,22].

In the present study, we carried out a search for an MHCI receptor that interacts with synaptic MHCI molecules and regulates the generation of asymmetries in hippocampal circuitry. MHCI itself does not initiate intracellular signaling cascades because its cytoplasmic tail is too short. Instead, MHCI signals by interacting with a variety of receptors [23,24]. One such MHCI receptor is paired immunoglobulin-like receptor B (PirB) [25–28]. Accumulating evidence indicates that PirB is expressed in subsets of neurons in the brain, including the cerebral cortex, hippocampus, cerebellum and olfactory bulb [29,30], and is localized at or near synapses [23,24,29]. Recent studies indicate that PirB inhibits axonal growth in cerebellar granule cells in response to myelin inhibitors [31], that it restricts ocular dominance (OD) plasticity following monocular deprivation (MD) in the visual cortex [29,32], and that it mediates the inhibitory effects of β-amyloid (Aβ) on hippocampal LTP [33]. Given these observations, we considered the possibility that MHCI signaling involved in the generation of hippocampal asymmetry might be transduced through PirB. Therefore, in the present study, we examined the effects of PirB deficiency on hippocampal asymmetry by performing electrophysiological and anatomical analyses on PirB knockout (KO) mice. We found that the functional and structural characteristics of PirB-deficient hippocampal synapses were very similar to those of ε2-dominant synapses, and furthermore, the hippocampal circuitry of PirB-deficient mice lacked both L–R and A–B asymmetries. These results, i.e. the lack of ε2-non-dominant synapses and the loss of circuit asymmetries in the PirB-deficient hippocampus, are fully consistent with our previous observations on the β2m-deficient hippocampus. Our findings suggest that the MHCI/PirB system plays a critical role in the generation of hippocampal asymmetry.

## Materials and methods

### Animals

C57BL/6 mice were purchased from Kyudo (Fukuoka, Japan). The PirB KO mice (C57BL/6 genetic background) were obtained as described previously [25]. The *iv* (SI/Col × C57BL/6J hybrid) mice were donated by Prof. H. Hamada (Osaka University, Japan). Animals were bred and maintained in the Animal Facility of the Faculty of Science, Kyushu University. Animals were monitored for disease every 6 months. The following were screened for: mouse hepatitis virus, Sendai virus, *Mycoplasma pulmonis*, Tyzzer’s organism, *Corynebacterium kutscheri*, *Citrobacter rodentium*, *Pasteurella pneumotropica*, *Salmonella* spp., intestinal protozoa, ectoparasites and pinworm. For the experiments, animals between 7 and 12 weeks of age (weighing about 20–25 g on average) were used. They appeared healthy until sacrifice. The experiments reported here were approved by the Animal Care and Use Committee of Kyushu University (No. A28-139-0). All experiments were performed according to the *Guideline for Animal Experiments* of the Faculty of Sciences, Kyushu University.

### Ventral hippocampal commissure (VHC) transection

To examine synapses made by ipsilateral Schaffer collateral fibers, the VHC was transected 5 days before electrophysiological recording [1,2,8,14]. Animals were anesthetized with a combination anesthetic, MMB, via intraperitoneal administration, and positioned with a stereotaxic apparatus. MMB was prepared with 0.75 mg/kg of medetomidine (Dorbene, Kyoritsuseiyaku Co., Ltd., Tokyo, Japan), 4.0 mg/kg of midazolam (Dormicum, Astellas Pharma Inc., Tokyo, Japan) and 5.0 mg/kg of butorphanol (Vetorphale, Meiji Seika Kaisha, Ltd., Tokyo, Japan) [34]. A small piece of razor blade (2.5 mm wide) was glued onto a rod that was clamped onto a micromanipulator. After removing a portion of the skull (3 mm wide and 4 mm long, including the bregma), the blade was inserted to a depth of 4.0 mm at the midline to transect the VHC. To avoid damaging the sagittal sinus, the blade was initially shifted 0.5 mm to the right and inserted 0.5 mm into the cerebral cortex, and was then returned to the midline position as the blade was lowered. After slowly removing the blade, the piece of skull was replaced, and the scalp was closed with sutures. Animals having undergone this procedure survived for over 3 months.

### Electrophysiology

Transverse hippocampal slices (450-μm-thick) were cut with a vibrating microtome (Linear Slicer PRO7, Dosaka EM, Kyoto, Japan) in ice-cold artificial cerebrospinal fluid (ACSF) (in mM: NaCl, 119; KCl, 2.5; CaCl_2_, 2.5; MgSO_4_, 1.3; NaH_2_PO_4_, 1.0; NaHCO_3_, 26; glucose, 10; saturated with 95% O_2_/5% CO_2_). Animals (7–12 weeks of age) were decapitated under deep sevoflurane anesthesia, and their brains were quickly removed. Brains were fixed on an agar block made of two pieces of agar (with a slope of 20°) adhered together at a right angle and mounted on the cutting stage. We lowered the left rear or right rear of the brain using the agar blocks when cutting the left or right hemisphere, respectively. Slices from a similar septotemporal level were used for the experiments. Recordings were made in a submerged slice chamber perfused with ACSF at 32 ± 1°C. Electrodes filled with 0.9% NaCl were used for extracellular recordings. Synaptic responses were evoked at 0.1 Hz using a bipolar tungsten electrode. The input–output relationship was examined by varying the intensity of the stimulation. To control for the differential recruitment of presynaptic axons in different slices, we plotted the field excitatory postsynaptic potential (fEPSP) amplitude against presynaptic fiber volley (PFV) amplitude. The amplitude of the PFV, when measured in ACSF, was very small compared with that of the fEPSP, and the measurement of very small responses is sometimes inaccurate. Therefore, we recorded under a moderately large PFV amplitude, obtained by using ASCF containing 5 mM Mg^2+^ and 0.7 μM 6,7-dinitroquinoxaline-2,3-dione (DNQX). Paired-pulse facilitation (PPF) was tested using a 50-ms interstimulus interval. Slices were perfused with ACSF. PPF values were calculated as the ratio of the second fEPSP peak divided by the first fEPSP peak. Synaptic plasticity-inducing stimuli were given at baseline stimulus strength. The fEPSP slope was expressed as a percentage of the mean slope value before the stimulations. Synaptic currents were recorded from CA1 or CA3 pyramidal neurons using the blind-patch technique [35] in the whole-cell voltage-clamp mode (Axopatch 1D, Axon Instruments, Foster City, CA, USA). A high-Mg^2+^/Ca^2+^ ACSF (4 mM MgSO_4_, 4 mM CaCl_2_) was used to increase membrane stability in the presence of bicuculline. Patch electrodes (4–6 MΩ) were filled with an intracellular solution (in mM: cesium gluconate, 122.5; CsCl, 17.5; Hepes buffer, 10; EGTA, 0.2; NaCl, 8; Mg-ATP, 2; Na_3_-GTP, 0.3; pH 7.2). We recorded non-NMDA EPSCs at a holding potential of −90 mV in the presence of bicuculline (30 μM). NMDA EPSCs were recorded at +30 mV (for measuring the NMDA EPSC/non-NMDA EPSC ratio) or +10 mV (for examining the inhibitory effects of Ro 25–6981) in the presence of DNQX (20 μM) and bicuculline (30 μM). We adopted a relatively low holding potential to obtain stable recordings of NMDA EPSCs in the Ro 25–6981 inhibition experiments [1,2,8,14]. The series resistance (10–30 MΩ) was regularly monitored during recordings. Cells were rejected if more than a 15% change in series resistance occurred during the experiment. All recordings were filtered at 2 kHz, digitized at 4 kHz, and stored on a computer equipped with an analog-to-digital converter (Power Lab 2/25, AD Instruments, Sydney, Australia). No failure was detected in our experiments. In input–output relationships, data were analyzed with the likelihood ratio test. In other experiments, data were expressed as means ± SEM and analyzed with Student’s *t*-test.

### Anatomy

For measurement of PSD size and ratio of perforated synapses, samples for electron microscopy (EM) were prepared as described previously [4]. In Brief, mice were deeply anesthetized with pentobarbital (60 mg/kg, i.p.) and transcardially perfused with 4% PFA + 0.05% glutaraldehyde in 0.1 M phosphate buffer, pH7.4. Thereafter, 50-μm-thick brain sections were cut and stained using osmium tetroxide and uranyl acetate, to provide adequate contrast for EM analysis. Samples were then infiltrated with and embedded in Durcupan resin. Following this, the middle one third of the stratum radiatum in the CA1 area was trimmed and exposed, and serial sections of 70 nm were obtained using an ultramicrotome (UCT, Leica). Serial images were taken from the sample under a transmission electron microscope (Tecnai 10, FEI) at 12,500× magnification. The images were analyzed using Reconstruct software [36]. Using the first image as a reference and the following images for the actual analysis, only newly appearing synapses were analyzed. We analyzed the PSD area of these synapses by measuring the length of the PSD on each image, then multiplying the length by the thickness of the section, and noting if they were perforated or not.

## Results

### Basic synaptic transmission profiles of hippocampal CA1 pyramidal neuron synapses in PirB-deficient mice

**Fig 2:**
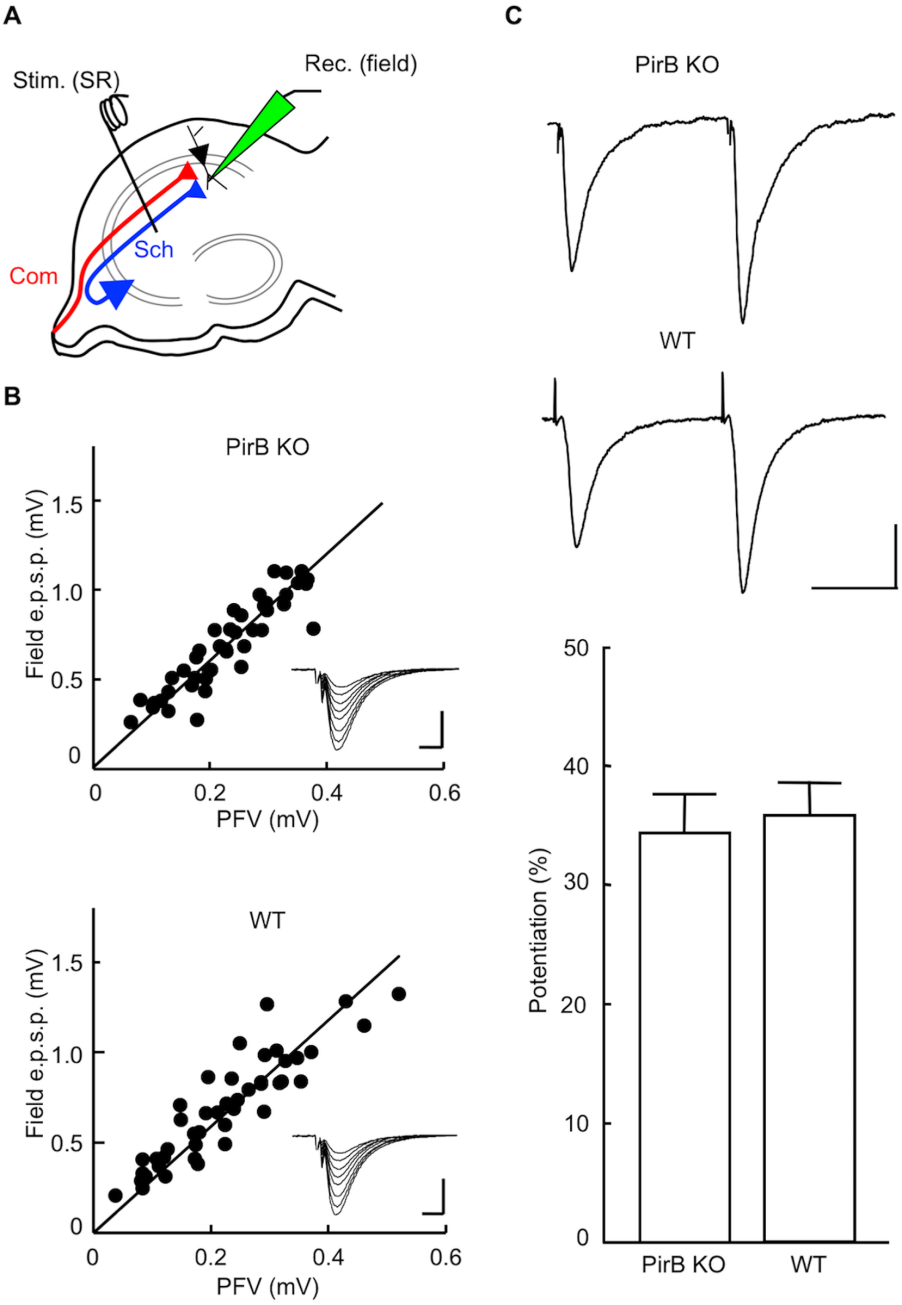
Targeted disruption of the *Pirb* gene does not alter basal synaptic transmission. (A) Schematic diagrams showing the arrangement of electrodes for extracellular recording. Using hippocampal slices prepared from naïve mice, fEPSPs were recorded with an extracellular electrode [Rec. (field)] placed in the stratum radiatum of area CA1. To activate apical synapses, a stimulating electrode was placed in the stratum radiatum of area CA1 [Stim. (SR)]. Sch, Schaffer collateral fibers; Com, commissural fibers. (B) Input–output relationship of fEPSP amplitude as a function of PFV amplitude in PirB KO mice (*n* = 8) and WT mice (*n* = 8). Inset traces show typical fEPSPs obtained with various stimulus intensities. Scale bars: 0.5 mV (vertical) and 5 ms (horizontal). (C) PPF induced by pairs of stimulus pulses delivered at interpulse intervals of 50 ms. Each trace is the average of three consecutive recordings. Scale bar: 0.5 mV (vertical) and 25 ms (horizontal). PPFs are expressed as the ratio of the second fEPSP divided by the first fEPSP (mean ± SEM; *n* = 11 each; *P* > 0.05, Student’s *t*-test).

It has been reported that targeted disruption of the *Pirb* gene does not influence the input–output relationship or PPF at Schaffer collateral–CA1 synapses [30]. Prior to the experiments on the PirB-deficient hippocampus, we reconfirmed the effect of PirB disruption on basic synaptic transmission at hippocampal CA1 pyramidal neuron synapses and obtained essentially the same results as those reported previously. First, we assessed the input–output relationship at a number of different stimulus intensities. To measure fEPSPs and PFVs, extracellular recording and stimulating electrodes were placed in the stratum radiatum of area CA1 (Fig 2A). Our analysis revealed no significant differences in the input–output relationship between PirB KO and WT mice (slope of input–output plot: PirB KO, 2.98, *n* = 8 from 8 animals; WT, 2.95, *n* = 8 from 8 animals; *P* > 0.05, likelihood ratio test; Fig 2B).

Next, to assess presynaptic function, we examined PPF at an interstimulus interval of 50 ms. We observed no difference in PPF at apical synapses of CA1 pyramidal neurons between slices from PirB KO and WT mice (PirB KO, 34.3 ± 3.4%, *n* = 11 from 7 animals; WT, 35.8 ± 2.8%, *n* = 11 from 7 animals; *P* > 0.05, *t*-test; Fig 2C).

### Inhibitory effect of Ro 25–6981 on NMDA EPSCs at CA1 pyramidal neuron synapses

**Fig 3:**
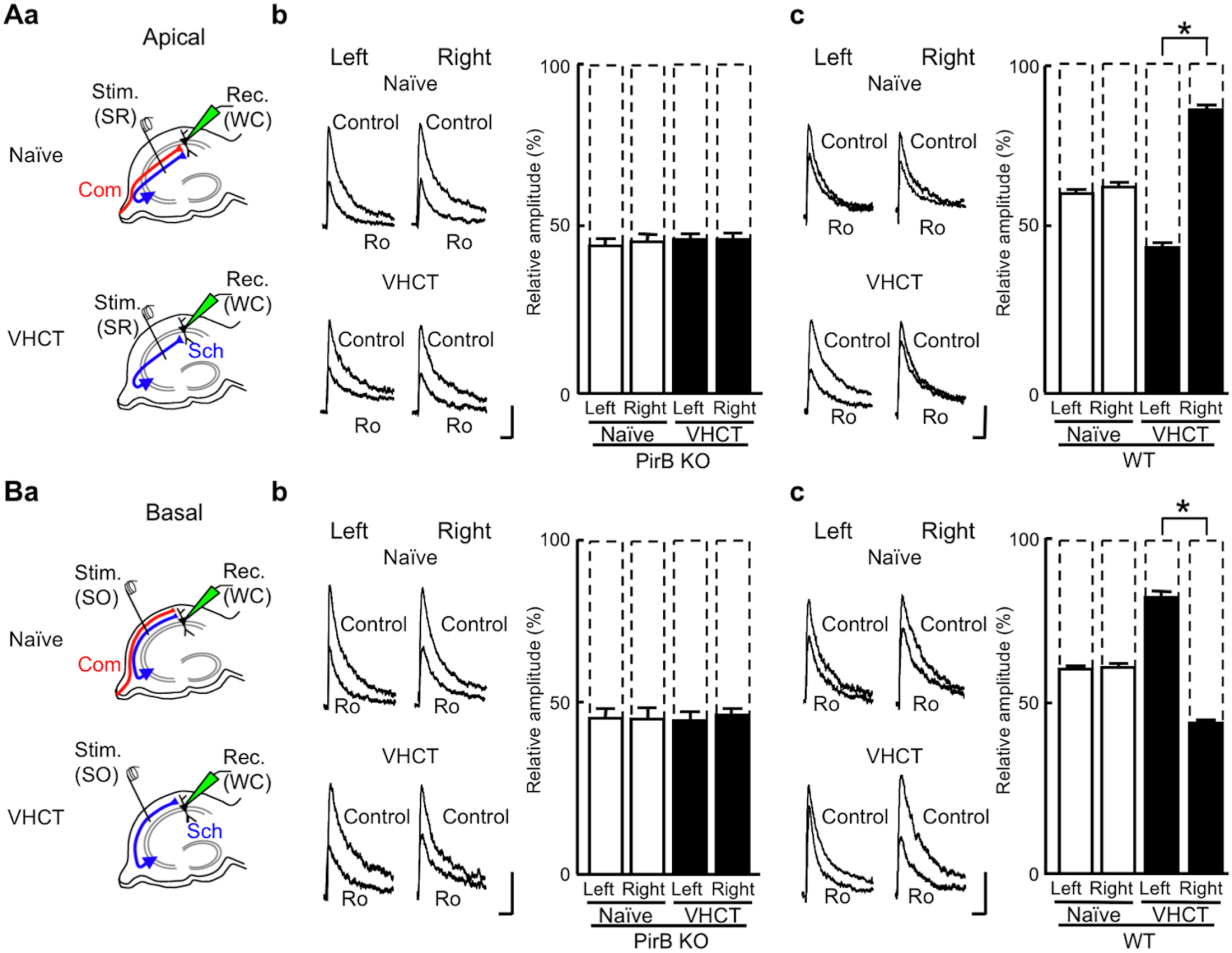
Inhibitory effect of Ro 25–6981 on NMDA EPSCs at CA1 pyramidal neuron synapses. (Aa) Schematic diagrams showing synaptic inputs onto the apical dendrites of CA1 pyramidal neurons and the positioning of electrodes. In slices from naïve and VHCT mice, electrical stimuli applied at the stratum radiatum [Stim. (SR)] of area CA1 selectively activated apical synapses. Whole-cell recordings [Rec. (WC)] were made from CA1 pyramidal neurons. Sch, Schaffer collateral fibers; Com, commissural fibers. (Ab) Effects of Ro 25–6981 on NMDA EPSCs at CA1 apical synapses in the PirB KO hippocampus. Representative superimposed traces indicate NMDA EPSCs recorded in the absence (Control) or presence of Ro 25–6981 (Ro, 0.6 μM). The levels of inhibition were maximal after exposure to Ro 25–6981 for 50 to 60 min. “Left” and “Right” indicate recordings from left and right hippocampal slices, respectively. Each trace is the average of five consecutive recordings. Scale bars: 25 pA (vertical) and 100 ms (horizontal). Relative amplitudes of NMDA EPSCs in the presence of Ro 25–6981 are expressed as percentages of control responses. Error bars represent SEM (*n* = 7 each; *P* > 0.05 for all combinations, Student’s *t*-test). (Ac) Effects of Ro 25–6981 on NMDA EPSCs at CA1 apical synapses in the WT hippocampus. Relative amplitudes of NMDA EPSCs in the presence of Ro 25–6981 are expressed as percentages of control responses (mean ± SEM; *n* = 7 each; *P < 0.05; absence of an asterisk indicates *P* > 0.05). (Ba) Schematic diagrams showing synaptic inputs onto the basal dendrites of CA1 pyramidal neurons and the positioning of electrodes. Whole-cell recordings [Rec. (WC)] were made from CA1 pyramidal neurons. To activate basal synapses, electrical stimuli were applied at the stratum oriens [Stim. (SO)] of area CA1. (Bb) Effects of Ro 25–6981 on NMDA EPSCs at CA1 basal synapses in the PirB KO hippocampus. Representative superimposed traces indicate NMDA EPSCs recorded in the absence (Control) or presence of Ro 25–6981 (Ro, 0.6 μM). “Left” and “Right” indicate recordings from left and right hippocampal slices, respectively. Scale bars: 25 pA (vertical) and 100 ms (horizontal). Relative amplitudes of NMDA EPSCs in the presence of Ro 25–6981 are expressed as percentages of control responses (mean ± SEM; *n* = 7 each; *P* > 0.05 for all combinations). (Bc) Effects of Ro 25–6981 on NMDA EPSCs at CA1 basal synapses in the WT hippocampus. Relative amplitudes of NMDA EPSCs in the presence of Ro 25–6981 are expressed as percentages of control responses (mean ± SEM; *n* = 7 each; *P < 0.05; absence of an asterisk indicates *P* > 0.05).

We examined the effects of Ro 25–6981 on NMDA EPSCs using hippocampal slices prepared from WT and PirB KO mice. To measure NMDA EPSCs, whole-cell recordings were made from CA1 pyramidal neurons (Fig 3) in the presence of DNQX (20 μM) and bicuculline (30 μM) at a holding potential of +10 mV [1]. To discriminate between excitatory synapses on the apical and basal dendrites of CA1 pyramidal neurons, NMDA EPSCs were elicited by electrical stimuli applied to the stratum radiatum (Fig 3A) or the stratum oriens (Fig 3B), respectively, of area CA1. Hippocampal CA1 pyramidal neurons receive major excitatory inputs from Schaffer collateral fibers originating from ipsilateral CA3 pyramidal neurons and commissural fibers from contralateral CA3 pyramidal neurons. To selectively characterize the NMDA receptors at Schaffer–CA1 pyramidal neuron synapses, we used hippocampal slices prepared from ventral hippocampal commissure-transected (VHCT) mice, in which commissural fibers have been denervated [1,2] (see Methods). We first examined NMDA EPSCs in hippocampal slices from PirB KO mice. In apical synapses, Ro 25–6981 (0.6 μM) reduced the peak amplitude of NMDA EPSCs to a similar extent in the left and right hippocampal slices of both naïve and VHCT mice (naïve: left, 44 ± 2% of control, *n* = 7 from 7 animals; right, 45 ± 2% of control, *n* = 7 from 7 animals; VHCT: left, 46 ± 2% of control, *n* = 7 from 7 animals; right, 46 ± 2% of control, *n* = 7 from 7 animals; *P* > 0.05 for all combinations; Fig 3Ab). Likewise, Ro 25–6981 sensitivity of basal synapses in the left and right hippocampi was similar in both naïve and VHCT mice (naïve: left, 47 ± 3% of control, *n* = 7 from 7 animals; right, 46 ± 3% of control, *n* = 7 from 7 animals; VHCT: left, 46 ± 2% of control, *n* = 7 from 7 animals; right, 47 ±2% of control, *n* = 7 from 7 animals; *P* > 0.05 for all combinations; Fig 3Bb). We next examined the sensitivity of NMDA EPSCs to Ro 25–6981 in hippocampal slices from WT mice. In naïve WT mice, Ro 25–6981 reduced the peak amplitude of NMDA EPSCs to a similar extent in left and right hippocampal slices in both apical (naïve: left, 62 ± 1% of control, *n* = 7 from 6 animals; right, 63 ± 1% of control, *n* = 7 from 6 animals; *P* > 0.05; Fig 3Ac) and basal (naïve: left, 62 ± 1% of control, *n* = 7 from 4 animals; right, 63 ± 1% of control, *n* = 7 from 6 animals; *P* > 0.05; Fig 3Bc) synapses. In contrast, VHCT WT mice showed asymmetrical Ro 25–6981 sensitivity. In apical synapses, Ro 25–6981 reduced NMDA EPSCs more robustly in left hippocampal slices than in right hippocampal slices (VHCT: left, 45 ± 1% of control, *n* = 7 from 7 animals; right, 87 ± 1% of control, *n* = 7 from 6 animals; *P* < 0.05; Fig 3Ac). Conversely, in basal synapses, slices from the right hippocampus exhibited greater sensitivity to Ro 25–6981 than slices from the left hippocampus (VHCT: left, 83 ± 1% of control, *n* = 7 from 6 animals; right, 45 ± 1% of control, *n* = 7 from 7 animals; *P* < 0.05; Fig 3Bc). The results for WT mice contain data that we have published previously (*n* = 3 each) [8]. In VHCT slices, Schaffer–CA1 synapses are responsible for NMDA EPSCs. Thus, our results indicate that the Schaffer–CA1 synapses in PirB KO mice lack asymmetry in the sensitivity of NMDA EPSCs towards Ro 25–6981.

### Inhibitory effect of Ro 25–6981 on NMDA EPSCs at commissural–CA3 synapses

**Fig 4:**
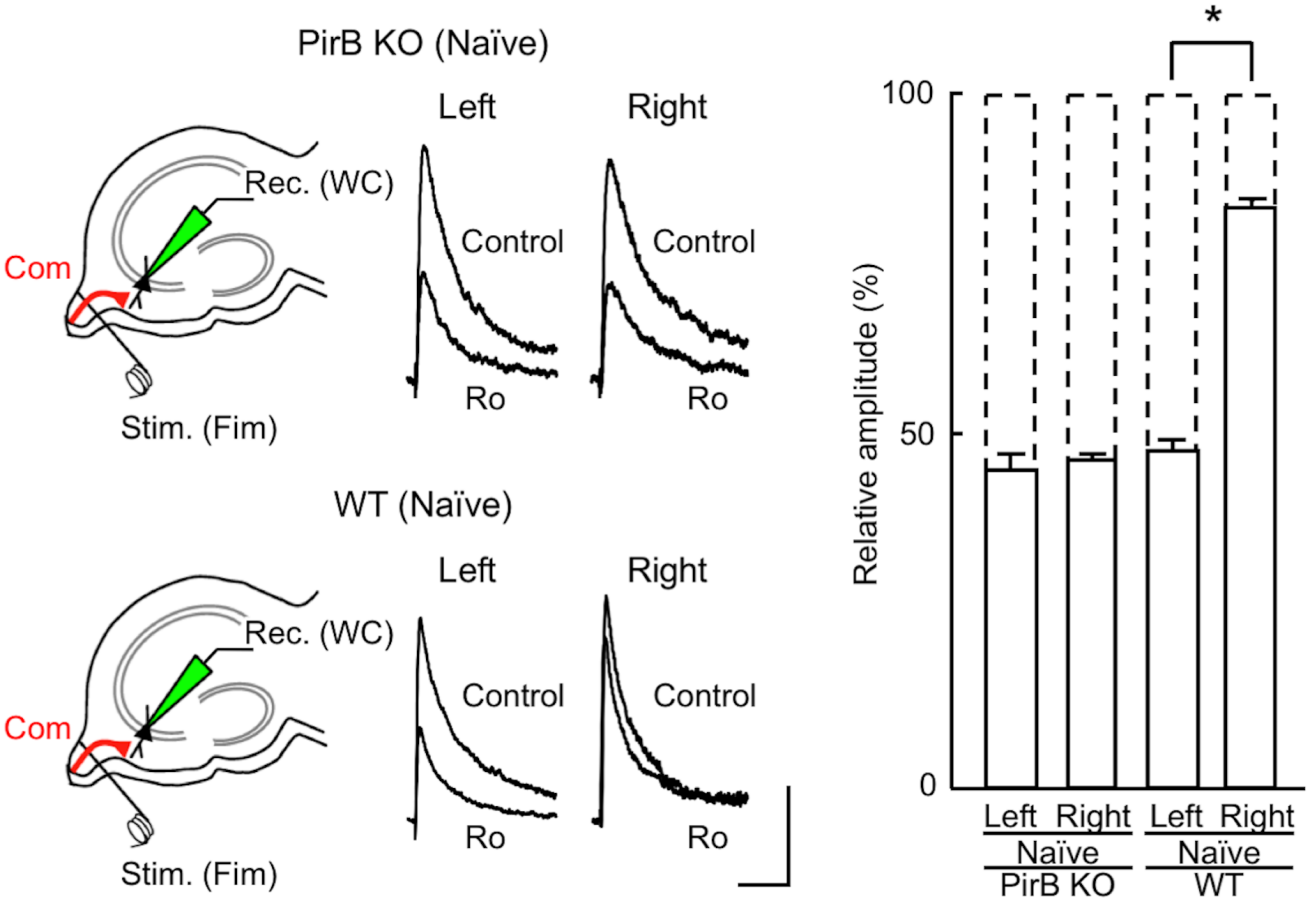
Inhibitory effects of Ro 25–6981 on NMDA EPSCs in the basal dendrites of CA3 pyramidal neurons. Schematic diagrams showing synaptic inputs onto the basal dendrites of CA3 pyramidal neurons in the PirB KO and WT mice. In slices from naïve mice, whole-cell recordings [Rec. (WC)] were made from CA3 pyramidal neurons. A stimulating electrode was placed in the ventral fimbria [Stim. (Fim)] to activate commissural fibers (Com). Representative superimposed traces indicate NMDA EPSCs recorded in the absence (Control) or presence of Ro 25–6981 (Ro, 0.6 μM). “Left” and “Right” indicate recordings from left and right hippocampal slices, respectively. Each trace is the average of five consecutive recordings. Scale bars: 25 pA (vertical) and 200 ms (horizontal). Relative amplitudes of NMDA EPSCs in the presence of Ro 25–6981 are expressed as percentages of control responses (mean ± SEM; *n* = 7 each; *P < 0.05; absence of an asterisk indicates *P* > 0.05).

We found that Ro 25–6981 sensitivity in naïve PirB-deficient mice, which have both Schaffer collateral fiber and commissural fiber synapses, was similar to that in VHCT PirB-deficient mice that have Schaffer collateral fiber synapses only (Fig 3Ab and 3Bb). Thus, in PirB-deficient mice, the sensitivity of commissural fiber synapses to Ro 25–6981 is expected to be similar to that of Schaffer collateral fiber synapses. Because it is not possible to selectively stimulate commissural fibers in the CA1 area, we examined the sensitivity of NMDA EPSCs to Ro 25–6981 at commissural fiber synapses formed on the basal dendrites of CA3 pyramidal neurons by stimulating the ventral fimbria in hippocampal slices from naïve mice [1]. To reduce contamination from commissural fiber responses on the apical dendrites of CA3 pyramidal neurons and to avoid antidromic activation of ipsilateral CA3 axons during stimulation of the ventral fimbria, we optimized the cutting angles in the preparation of hippocampal slices [1] (see Methods). Consistent with our previous results obtained using the WT hippocampus [1,8], Ro 25–6981 reduced NMDA EPSCs to a greater extent in left commissural–CA3 synapses than in right synapses (left, 48 ± 2% of control, *n* = 7 from 6 animals; right, 83 ± 1% of control, *n* = 7 from 6 animals; *P* < 0.05; Fig 4, WT). In PirB-deficient mice, however, Ro 25–6981 reduced the peak amplitude of NMDA EPSCs at commissural–CA3 synapses to a similar extent in left and right hippocampal slices (left, 45 ± 2% of control, *n* = 7 from 7 animals; right, 47 ± 1% of control, *n* = 7 from 7 animals; *P* > 0.05; Fig 4, PirB KO), and the levels of inhibition were similar to those observed for Schaffer–CA1 synapses (Fig 3Ab and 3Bb, VHCT). These results, obtained using naïve mice, suggest that Ro 25–6981 sensitivity of NMDA EPSCs in the PirB-deficient hippocampus is similar for Schaffer and commissural fiber synapses, and that the lack of asymmetry in the PirB-deficient hippocampus is not caused by VHCT. Together, these observations suggest that the hippocampal circuitry of PirB-deficient mice lacks both L–R and A–B asymmetries in the sensitivity of NMDA EPSCs to Ro 25–6981.

### Amplitude of NMDA EPSCs at hippocampal CA1 pyramidal neuron synapses in PirB-deficient mice

**Fig 5:**
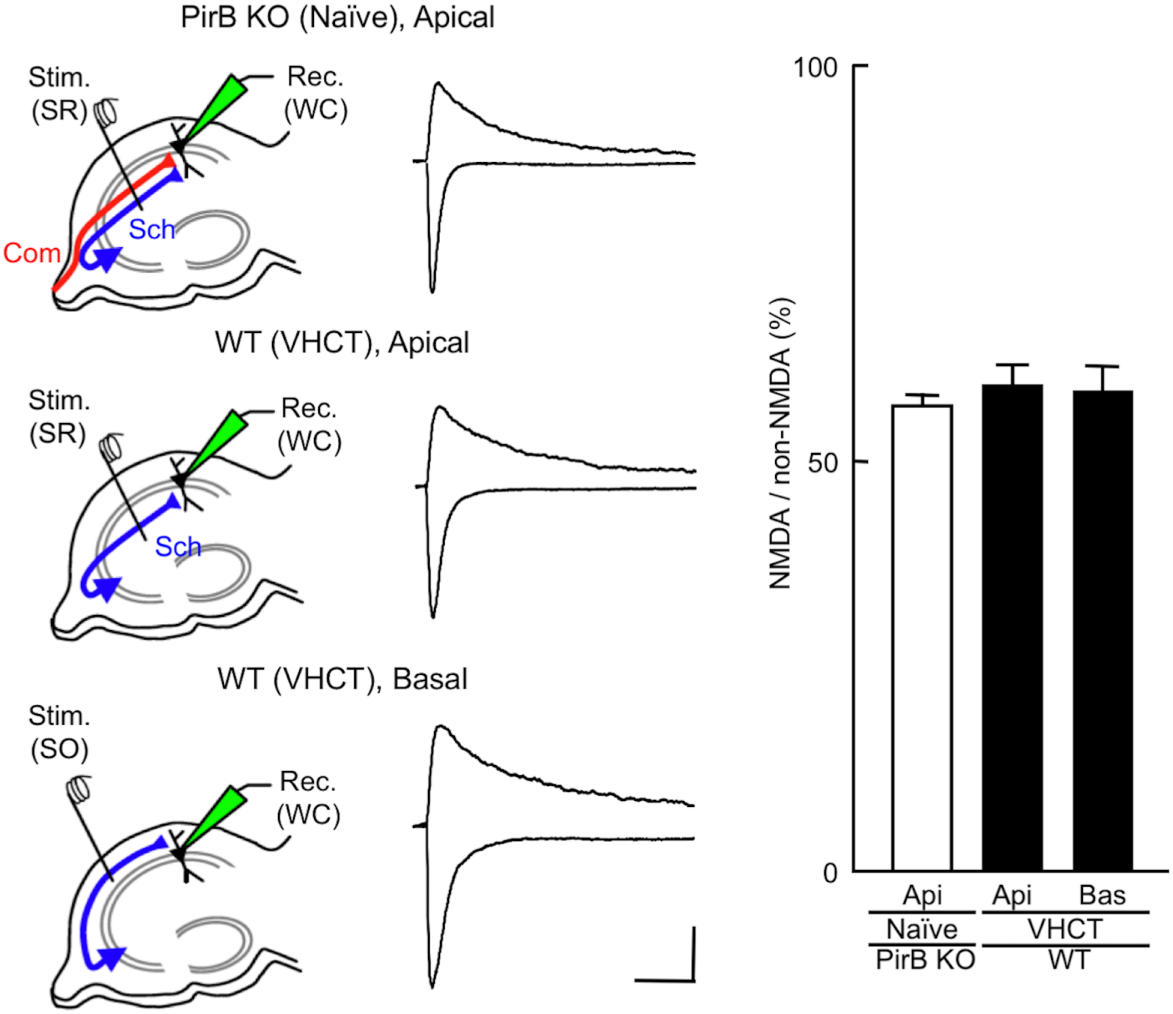
Effect of PirB deficiency on the amplitude of NMDA EPSCs evoked in CA1 pyramidal neuron synapses. Schematic diagrams showing the arrangement of electrodes for recording. A stimulating electrode was placed in the stratum radiatum [Stim. (SR)] or the stratum oriens [Stim. (SO)] of the CA1 to activate apical or basal synapses, respectively. Whole-cell patch recordings [Rec. (WC)] were made from CA1 pyramidal neurons. Sample superimposed traces of representative EPSCs recorded in hippocampal slices prepared from PirB KO and WT mice. The top traces show NMDA EPSCs at +30 mV in the presence of DNQX and bicuculline. The bottom traces show non-NMDA EPSCs at −90 mV in the presence of bicuculline. Each trace was averaged from five consecutive recordings. Scale bars: 50 pA (vertical) and 100 ms (horizontal). Relative amplitudes of NMDA EPSCs are expressed as percentages of non-NMDA EPSCs. Columns and error bars represent means and SEM, respectively (*n* = 7 each; *P* > 0.05, *t*-test). Api, apical synapses; Bas, basal synapses.

We next examined the amplitudes of NMDA EPSCs at CA1 pyramidal neuron synapses in hippocampal slices prepared from PirB KO and VHCT WT mice. Whole-cell recordings were made from CA1 pyramidal neurons in slices prepared from the left hippocampus. The amplitudes of NMDA EPSCs were expressed as a ratio to the amplitudes of non-NMDA EPSCs evoked at the same stimulation intensity. Non-NMDA EPSCs were recorded at a holding potential of −90 mV in the presence of bicuculline (30 μM). NMDA EPSCs were measured at +30 mV in the presence of DNQX (20 μM) and bicuculline (30 μM). The amplitudes of NMDA EPSCs, estimated by the NMDA/non-NMDA EPSC ratios, were similar for ε2-dominant and ε2-non-dominant synapses in WT mice, and for PirB KO and WT mice (naïve PirB KO, apical synapse, 57 ± 2%, *n* = 7 from 7 animals; VHCT WT, apical, ε2-dominant, 61 ± 2%, *n* = 7 from 6 animals; VHCT WT, basal, ε2-non-dominant, 60 ± 2%, *n* = 7 from 7 animals; *P* > 0.05 for all combinations, *t*-test, Fig 5).

### Comparison of the Ro 25–6981 sensitivity of NMDA EPSCs in PirB KO and *iv* mice

**Fig 6:**
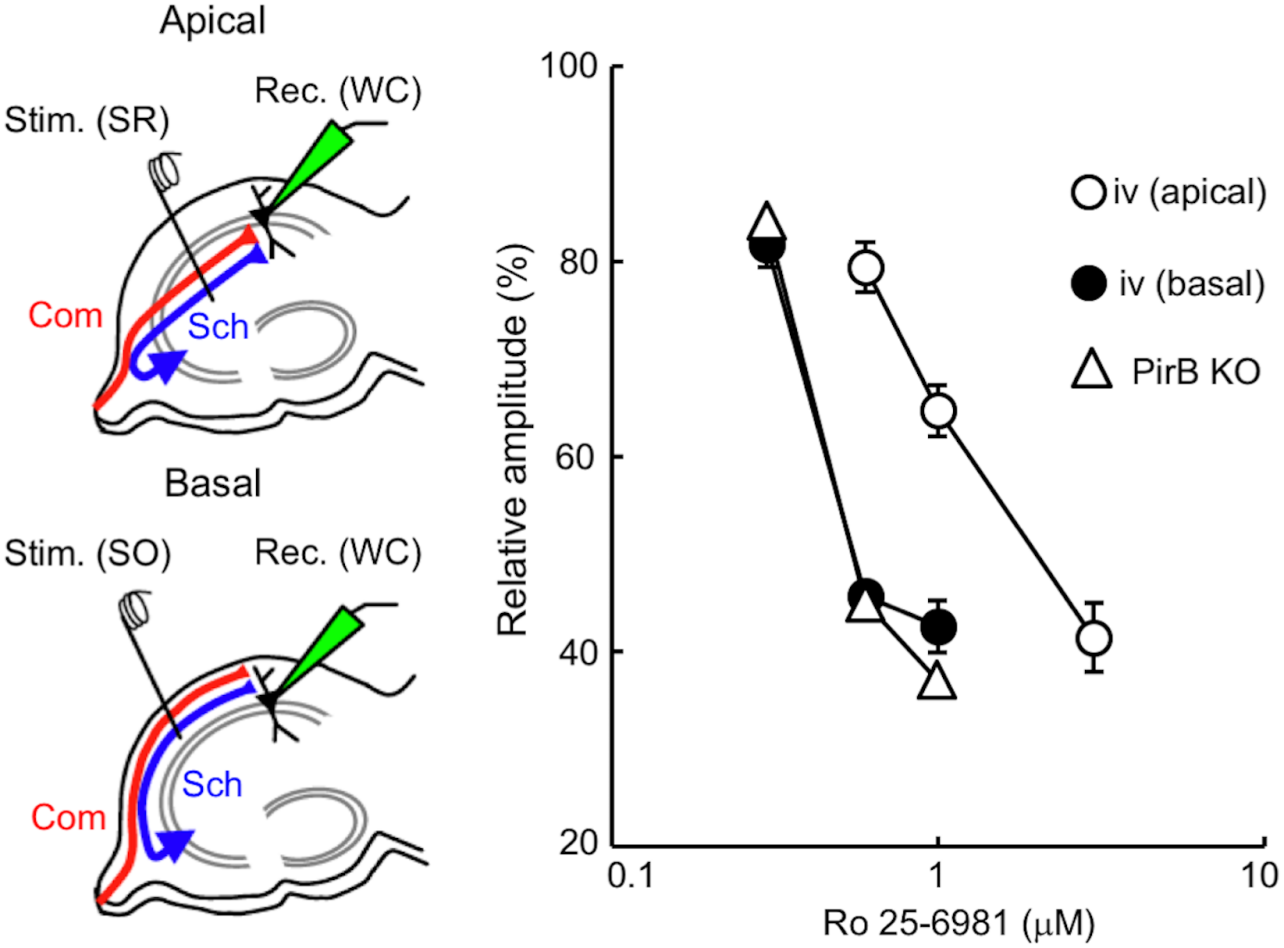
Comparison of the concentration dependency of Ro 25–6981 inhibition of NMDA EPSCs in hippocampal synapses of PirB KO and *iv* mice. Schematic diagrams showing synaptic inputs onto the apical and basal dendrites of CA1 pyramidal neurons and the arrangement of electrodes. In hippocampal slices prepared from naïve mice, whole-cell recordings [Rec. (WC)] were made from CA1 pyramidal neurons. A stimulating electrode was placed in the stratum radiatum [Stim. (SR)] or the stratum oriens [Stim. (SO)] of the CA1 to activate apical or basal synapses, respectively. Sch, Schaffer collateral fibers; Com, commissural fibers. NMDA EPSCs were recorded at a holding potential of +10 mV. Relative amplitudes of NMDA EPSCs in the presence of several concentrations of Ro 25–6981 are expressed as percentages of control responses (mean ± SEM). Filled and open circles represent basal [iv (basal)] and apical [iv (apical)] synapses (*n* = 7 each), respectively, of CA1 pyramidal neurons in the *iv* mouse hippocampus. Open triangles represent CA1 apical synapses in the PirB KO hippocampus (*n* = 10 each).

To evaluate the Ro 25–6981 sensitivity of PirB-deficient hippocampal synapses, we compared the dose–response characteristics of Ro 25–6981 inhibition of NMDA EPSCs in PirB KO and *iv* mice. In hippocampal slices from naïve *iv* mice, electrical stimuli were applied at the stratum radiatum to record NMDA EPSCs at apical synapses, while they were applied at the stratum oriens to record NMDA EPSCs at basal synapses (Fig 6). In hippocampal slices from naïve PirB KO mice, NMDA EPSCs were elicited in apical synapses of CA1 pyramidal neurons in response to electrical stimuli applied at the stratum radiatum. We analyzed left and right hippocampal slices (*n* = 7 and *n* = 3, respectively) prepared from PirB KO mice, and the results were combined because there were no significant differences between the two groups. In the *iv* hippocampus, the dose–response relationship was shifted towards the left in basal synapses compared with apical synapses and was almost the same as that obtained in hippocampal synapses of the PirB KO (Fig 6). This indicates that the Ro 25–6981 sensitivity of hippocampal synapses in the PirB KO is similar to that of ε2-dominant synapses in the *iv* hippocampus.

### Relationship between stimulation frequency and synaptic plasticity

**Fig 7:**
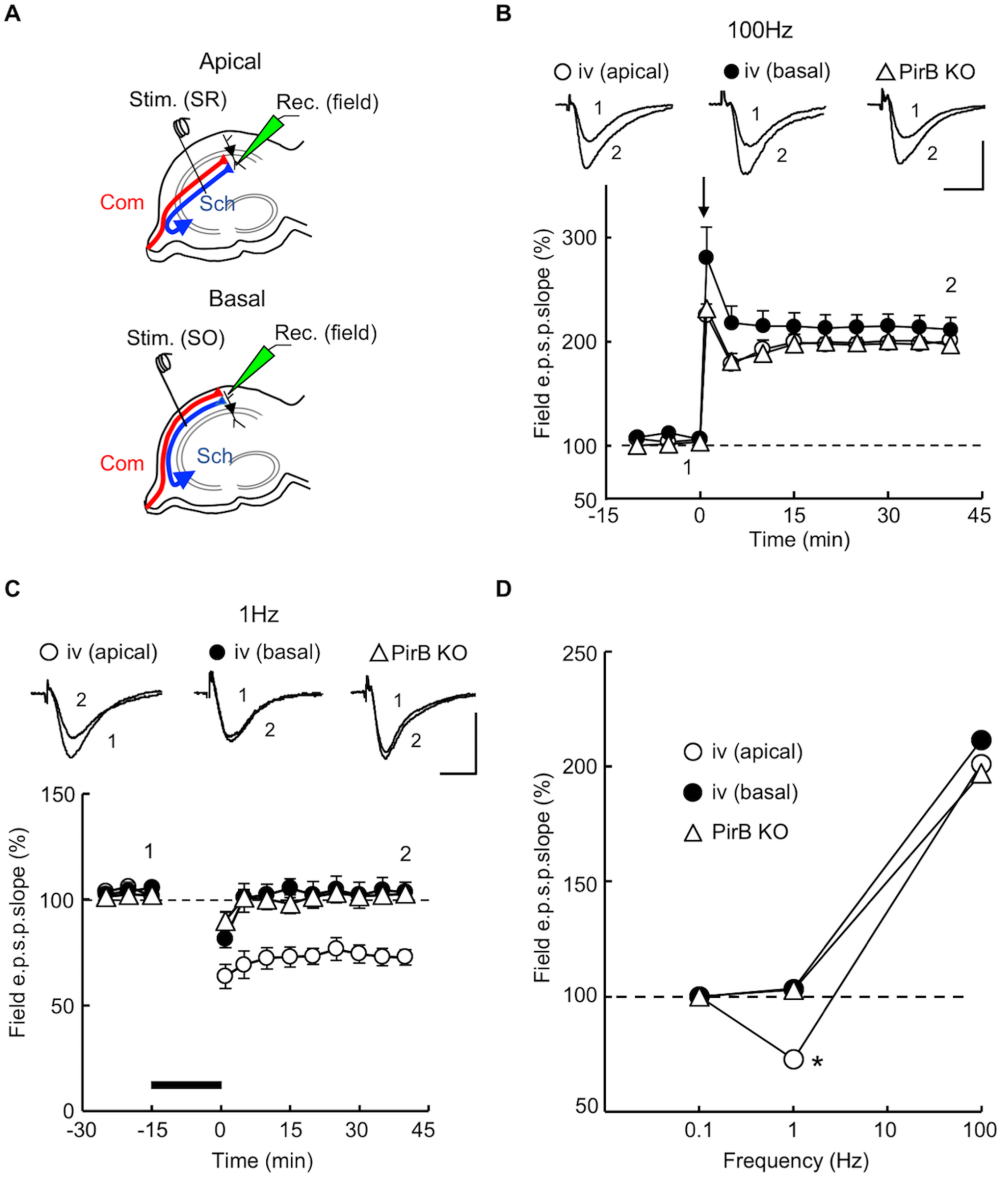
Relationship between synaptic plasticity and stimulation frequency in hippocampal synapses of PirB KO and *iv* mice. (A) Schematic diagrams of the arrangement of electrodes for extracellular recording. Using hippocampal slices prepared from naïve mice, fEPSPs were recorded with an extracellular electrode [Rec. (field)] placed either in the stratum radiatum or stratum oriens of the CA1. To activate apical (Apical) or basal (Basal) dendritic synapses, a stimulating electrode was placed in the stratum radiatum [Stim. (SR)] or stratum oriens [Stim. (SO)], respectively. (B) Tetanic stimulation (100 Hz for 1 s, 3 trains, interval of 10 s) applied at time 0 (arrow) elicited LTPs of the fEPSP slope in hippocampal slices from both PirB KO and *iv* mice. Filled and open circles represent, respectively, basal [iv (basal), *n* = 7] and apical [iv (apical), *n* = 7] synapses of CA1 pyramidal neurons in the *iv* mouse hippocampus. Open triangles represent CA1 apical synapses in the PirB KO hippocampus (*n* = 10). Symbols and error bars represent means and SEM, respectively. The upper superimposed traces show representative fEPSPs recorded before (1) or 40 min after (2) tetanic stimulation. Scale bars: 1.0 mV (vertical) and 10 ms (horizontal). (C) Low-frequency stimulation (1 Hz for 15 min, thick bar) induced LTD in apical synapses of the *iv* mouse hippocampus (open circles, *n* = 7), but not in basal synapses of the *iv* mouse hippocampus (filled circles, *n* = 7) or in PirB KO synapses (open triangles, *n* = 10). The upper superimposed traces are representative fEPSPs recorded before (1) or 40 min after (2) low-frequency stimulation. Scale bars: 1.0 mV (vertical) and 10 ms (horizontal). (D) Stimulation frequency dependency of synaptic plasticity. Relative amplitudes of fEPSP slopes, estimated 40 min after tetanus, were plotted against stimulation frequency (mean ± SEM). Points at 0.1 Hz (test pulse frequency) indicate baseline values (horizontal dashed line). Symbols are the same as those in (B) and (C). *P < 0.05.

Synaptic plasticity in the hippocampus is dependent on stimulation frequency [37–39], and therefore, we compared the stimulation frequency dependency of synaptic plasticity in PirB KO and *iv* mice. Hippocampal slices were perfused with ACSF, and maintained at 32 ± 1°C. In slices from naïve *iv* mice, the stimulating electrode was placed in the stratum radiatum or the stratum oriens to measure CA1 pyramidal neuron fEPSPs at apical or basal synapses, respectively (Fig 7A) [8,14]. In slices from naïve PirB KO mice, the stimulating electrode was placed in the stratum radiatum, and fEPSPs evoked on the apical dendrites of CA1 pyramidal neurons were recorded (Fig 7A). We examined both left and right hippocampal slices (*n* = 7 and *n* = 3, respectively) prepared from PirB KO mice, and the results were combined because there were no significant differences between the two groups. The baseline stimulus intensity was adjusted to elicit fEPSPs with an amplitude of about a third of the maximum response. Synaptic plasticity-inducing stimuli were given at baseline stimulus strength. In hippocampal slices from adult *iv* mice, tetanic stimulation (100 Hz for 1 s, 3 trains, interval of 10 s) induced LTP of the slope of the fEPSP in both apical and basal synapses of CA1 pyramidal neurons (relative fEPSP slope 40 min after tetanic stimulation: apical synapses, 203 ± 7% of control, *n* = 7 from 7 animals; basal synapses, 213 ± 12% of control, *n* = 7 from 6 animals; Fig 7B and 7D). As has been reported previously [30], hippocampal slices from adult PirB KO mice showed LTP in response to the same tetanus (relative fEPSP slope 40 min after tetanic stimulation: 197 ± 5% of control, *n* = 10 from 10 animals; Fig 7B and 7D). No significant difference (*P* > 0.05) was observed in the amplitude of LTP among these three populations of synapses.

The delivery of low-frequency stimulation (1 Hz for 15 min) induced no detectable change in the fEPSP slope in basal synapses in *iv* mice (103 ± 5% of control, *n* = 7 from 6 animals) or in the PirB KO (103 ± 2% of control, *n* = 10 from 10 animals), whereas significant long-term depression (LTD) was detected in apical synapses in *iv* mice (73 ± 4% of control, *n* = 7 from 6 animals, *P* < 0.05; Fig 7C and 7D). No significant difference was observed between *iv* basal and PirB KO synapses (Fig 7C and 7D). Although a robust LTD in response to low-frequency stimulation (1 Hz for 15 min) has been reported for hippocampal slices prepared from juvenile PirB KO mice (postnatal days 15–17) [30], no significant LTD was detected in hippocampal slices from adult PirB KO mice (7–12 weeks of age). Our results indicate that the relationship between stimulation frequency and synaptic plasticity in adult synapses in the PirB KO is very similar to that of the ε2-dominant synapse in the *iv* mouse.

### Morphology of dendritic spine synapses in the PirB KO mouse hippocampus

**Fig 8:**
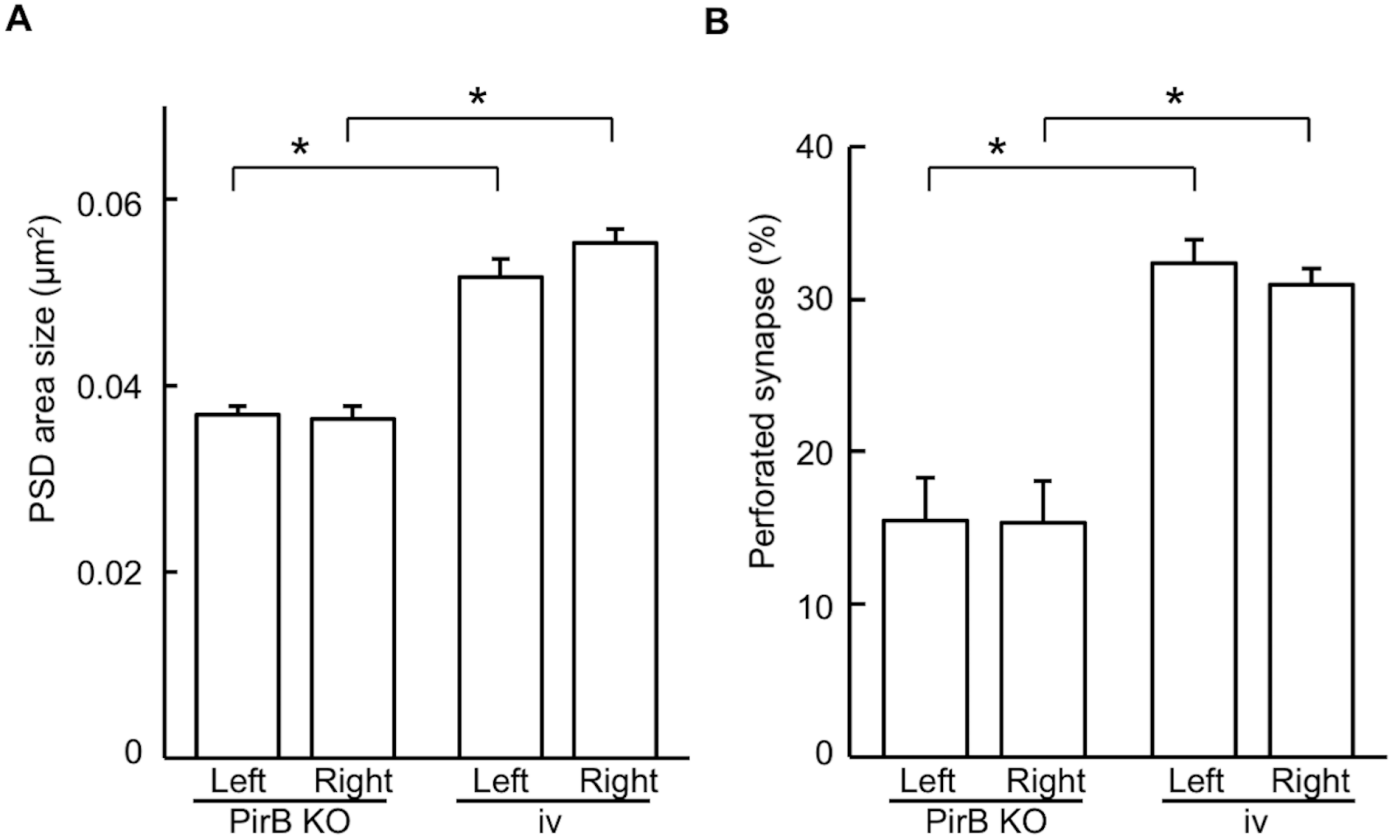
Laterality defects in hippocampal synapse morphology in PirB KO and *iv* mice. Postsynaptic density (PSD) area (A) and percentage of perforated synapses (B) were compared between left and right CA1 pyramidal cell synapses in the PirB KO and *iv* mice. No significant difference in these parameters between the left and right hippocampus was observed in either PirB KO or *iv* mice, whereas significant differences were found between PirB KO and *iv* hippocampi. Error bars represent s.e.m. An asterisk indicates *P* < 0.05; absence of an asterisk indicates *P* > 0.05.

In the CA1 stratum radiatum of wild-type mice, synapses that receive inputs from the right CA3 (ε2-non-dominant synapses) are larger, and with a higher ratio of perforated synapses, than synapses that receive inputs from the left CA3 (ε2-dominant synapses) [4]. Therefore, we next examined the morphology of synapses in PirB KO and *iv* mice and assessed whether they correspond to ε2-non-dominant or ε2-dominant synapses in wild-type mice. To this end, we evaluated PSD area and the ratio of perforated synapses in CA1 apical dendritic synapses by electron microscopy. In PirB KO mice, no significant difference in the size of the PSD area or the ratio of perforated synapses was detected between the left and right hippocampus (PSD area: left, 0.0369 ± 0.0009 μm^2^, *n* = 116 from 3 animals; right, 0.0364 ± 0.0014 μm^2^, *n* = 117 from 3 animals, *P* > 0.05; Fig 8A; perforated synapse ratio: left, 15.4 ± 2.8%, *n* = 116 from 3 animals; right, 15.3 ± 2.7%, *n* = 117 from 3 animals, *P* > 0.05; Fig 8B). Similarly, in *iv* mice, both of these ultrastructural parameters were comparable between the left and right hippocampus (PSD area: left, 0.0517 ± 0.0020 μm^2^, *n* = 96 from 3 animals; right, 0.0554 ± 0.0015 μm^2^, *n* = 103 from 3 animals, *P* > 0.05; Fig 8A; perforated spine ratio: left, 32.4 ± 2.3%, *n* = 96 from 3 animals; right, 31.0 ± 1.9%, *n* = 103 from 3 animals, *P* > 0.05; Fig 8B). However, the values of these parameters were larger in *iv* hippocampal synapses than in PirB KO hippocampal synapses (*P* < 0.05; Fig 8). Consistent with the ε2 dominance, the size of the PSD area and the ratio of perforated synapses in *iv* and PirB KO mice were similar to those in the right-input (ε2-non-dominant) and left-input (ε2-dominant) synapses in wild-type mice [4]. Furthermore, these parameters were also similar between PirB KO and β2m KO mice [8].

## Discussion

**Fig 9:**
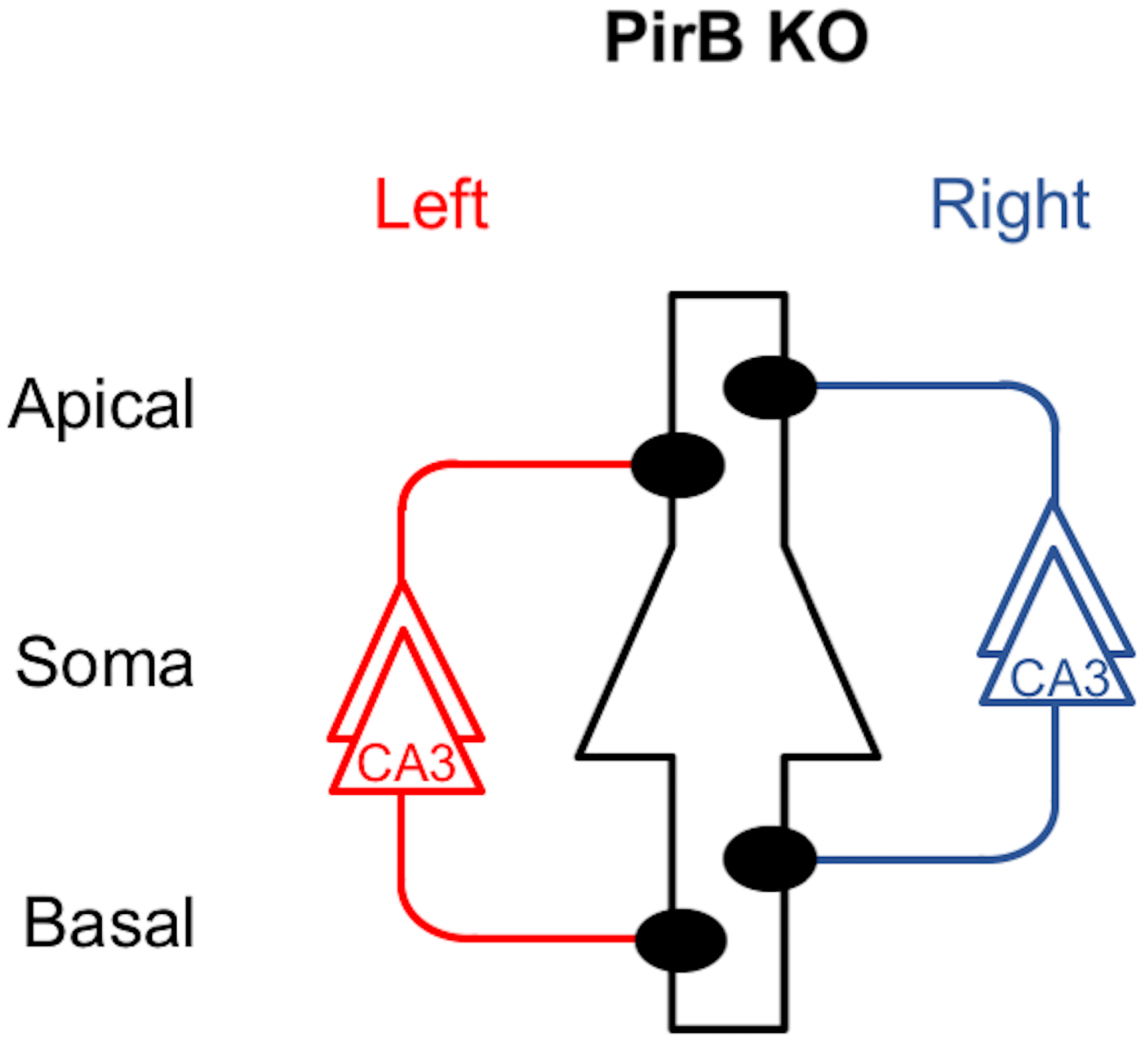
Asymmetry defects in the PirB KO mouse hippocampus. A postsynaptic CA1 pyramidal neuron is in the center, outlined in black, and it represents postsynaptic neurons in both left and right hemispheres. Left and right CA3 pyramidal neurons and their axons are colored red and blue, respectively. Filled circles represent ε2-dominant synapses. Note that ε2-non-dominant synapses are absent and circuit asymmetry is lost in the PirB KO hippocampus. This asymmetry defect is the same as that in the β2m KO hippocampus (shown in Fig 1).

To investigate the role of PirB in the generation of hippocampal asymmetries, we analyzed PirB KO mice. We found that the PirB-deficient hippocampus lacks both L–R and A–B asymmetries in neuronal circuitry. Our results demonstrate that basic synaptic transmission in hippocampal CA1 pyramidal neuron synapses, assessed by the input–output relationship and PPF, was similar in PirB KO and WT mice (Fig 2). The Ro 25–6981 sensitivity of NMDA EPSCs at PirB-deficient synapses was indistinguishable between the left and right hippocampus or between the apical and basal synapses of pyramidal neurons for both Schaffer and commissural fiber synapses (Figs 3 and 4). The amplitudes of NMDA EPSCs, estimated by the NMDA/non-NMDA EPSC ratio, were also similar among the PirB KO and WT mice (Fig 5). The dose–response characteristics of Ro 25–6981 inhibition of NMDA EPSCs and the stimulation frequency dependency of synaptic plasticity in PirB-deficient synapses were very similar to those of the ε2-dominant synapse in the *iv* mouse (Figs 6 and 7). In addition, morphological analysis (i.e., measurement of the area of the PSD and the ratio of perforated synapses) revealed that PirB KO synapses lacked L–R asymmetry and were comparable to β2m KO hippocampal synapses and ε2-dominant synapses in WT mice (Fig 8). Taken together, the results of these experiments suggest that the PirB-deficient mouse hippocampus lacks ε2-non-dominant synapses, containing ε2-dominant synapses only, resulting in a total loss of circuit asymmetry (Fig 9). This phenotypic feature of the PirB KO is identical to that of the β2m KO (see Fig 1) [8], suggesting that the MHCI signaling that generates hippocampal asymmetry is transduced through PirB. Furthermore, the observation that functional pyramidal neuron synapses are formed in both β2m-deficient and PirB-deficient mice indicates that MHCI signaling through PirB is critical for the generation of circuit asymmetry, but is dispensable for synapse formation *per se*.

It has been reported that the delivery of low-frequency stimulation (1Hz for 15 min) induces LTD at CA1 pyramidal neuron synapses in hippocampal slices prepared from PirB-deficient mice [30]. However, neither LTD nor LTP were detected in our present study. This discrepancy could be owing to differences in the age of the animals used and/or the concentration of Mg^2+^ in the ACSF. In our present study, we prepared hippocampal slices from adult PirB KO mice (7–12 weeks of age) and perfused these with normal ACSF containing 2.5 mM Mg^2+^, whereas hippocampal slices from juvenile (P15–17) PirB KO pups and high-Mg^2+^ ACSF (5 mM Mg^2+^) were used in the study by the other group. In the adult mouse hippocampus, NMDA receptor GluRζ (NR1), GluRε1 (NR2A) and ε2 (NR2B) subunits are expressed [40,41]. Although the ζ and ε2 subunits are expressed in the mouse hippocampus throughout development, the ε1 subunit is expressed only after birth. Therefore, the amount of synaptic ε1 subunit is likely to be low in the juvenile hippocampus compared with the adult hippocampus. The lower levels of the ε1 subunit should result in a comparatively smaller number of functional NMDA receptors at juvenile hippocampal synapses. Both a decrease in the number of synaptic NMDA receptors and an increase in the concentration of Mg^2+^ in ACSF are likely to reduce the rise in cytoplasmic Ca^2+^ caused by low frequency stimulation-induced activation of NMDARs. Because a weak increase in cytoplasmic Ca^2+^ favors LTD induction [42], it may be possible to induce LTD under the conditions used by the other group.

The pre- and postsynaptic localization of the MHCI and PirB molecules that participate in the establishment of hippocampal asymmetry are unclear. Recent studies have shown that MHCI molecules are present in both axons and dendrites of neurons in culture [43,44], and *in vivo* in the rodent cortex [43–47]. In contrast, PirB proteins are detected in the growth cones of cortical neurons in culture [29]. However, it remains unknown whether these molecules are involved in asymmetry generation at those synapses. MHCI family members are expressed on all nucleated cells and have a high molecular diversity. Although more than 70 MHCI family members are known in rodents [48], only some of the members likely participate in the generation of hippocampal asymmetries. Furthermore, in the immune system, PirB binds to MHCI either in *cis*, i.e. on the same cell, or in *trans*, i.e. on another cell [49]; however, it is currently unknown whether PirB binds to MHCI in either configuration in the brain. To further clarify the role of MHCI/PirB signaling in the generation of hippocampal asymmetries, the pre- and postsynaptic localization of MHCI and PirB molecules will need to be elucidated.

In addition to MHCI, three myelin-associated growth inhibitory proteins—neurite outgrowth inhibitor protein (Nogo), myelin associated glycoprotein (MAG) and oligodendrocyte myelin glycoprotein (OMgp)—are endogenous PirB ligands [31]. Nogo is expressed in the hippocampus [30,50], visual cortex [51] and olfactory bulb [52], and is present at synapses [30]. Recent studies have revealed that Nogo regulates NMDAR-dependent LTP at hippocampal synapses [30,50,53] and ocular dominance plasticity following monocular deprivation in the visual cortex [51]. The high-affinity binding sites for MHCI and Nogo on the PirB ectodomain differ. MHCI binds only to the N-terminal domain of PIR-B, whereas Nogo binds with high affinity to the C-terminal ectodomain [54]. Therefore, MHCI and Nogo should be capable of binding to PirB simultaneously. It is currently not known whether Nogo has a role in the generation of hippocampal asymmetry. However, Nogo could possibly modulate MHCI/PirB signaling via interaction with PirB, thereby participating in the establishment of hippocampal circuitry asymmetry.

In conclusion, the PirB-deficient hippocampus lacks ε2-non-dominant synapses and circuit asymmetries. The present study, along with our previous studies of β2m-deficient mice, provides further evidence that the MHCI/PirB signaling pathway is critical for the generation of hippocampal asymmetry. Although many important features of the MHCI/PirB system in the brain remain to be explored, our present findings suggest that this signaling pathway has a critical function in the generation of defined neuronal circuits in the brain.
